# Unsupervised Retinal Vessel Segmentation Using Combined Filters

**DOI:** 10.1371/journal.pone.0149943

**Published:** 2016-02-26

**Authors:** Wendeson S. Oliveira, Joyce Vitor Teixeira, Tsang Ing Ren, George D. C. Cavalcanti, Jan Sijbers

**Affiliations:** 1 Centro de Informática, Universidade Federal de Pernambuco, Recife, PE, Brazil; 2 iMinds - Vision Lab, Department of Physics, University of Antwerp, Wilrijk, Belgium; Pennsylvania State Hershey College of Medicine, UNITED STATES

## Abstract

Image segmentation of retinal blood vessels is a process that can help to predict and diagnose cardiovascular related diseases, such as hypertension and diabetes, which are known to affect the retinal blood vessels’ appearance. This work proposes an unsupervised method for the segmentation of retinal vessels images using a combined matched filter, Frangi’s filter and Gabor Wavelet filter to enhance the images. The combination of these three filters in order to improve the segmentation is the main motivation of this work. We investigate two approaches to perform the filter combination: weighted mean and median ranking. Segmentation methods are tested after the vessel enhancement. Enhanced images with median ranking are segmented using a simple threshold criterion. Two segmentation procedures are applied when considering enhanced retinal images using the weighted mean approach. The first method is based on deformable models and the second uses fuzzy C-means for the image segmentation. The procedure is evaluated using two public image databases, Drive and Stare. The experimental results demonstrate that the proposed methods perform well for vessel segmentation in comparison with state-of-the-art methods.

## Introduction

Retinal vessel segmentation is an image processing procedure that can help in the detection of numerous eye diseases [1]. Complete and correct segmentation is normally required for proper analysis of the vessels and their branching patterns. Manual tracing of retinal vessels is one method that can be used for segmentation. However, it is a long and tedious task which also requires training and is prone to interoperator variability [2]. Automatic segmentation is desirable but is generally vulnerable to artifacts/noise, image resolution, illumination and other variations present in the images. Furthermore, the retinal images present two vascular networks: the arterial and the venous. These vessels cross and overlap with some frequency, especially next to the optical disc, hindering the automatic segmentation of the image. For these reasons, retinal vessel segmentation still poses a great challenge.

A wide variety of blood vessel segmentation algorithms are described in the literature. In general, these algorithms can be grouped into supervised and unsupervised methods. Supervised methods [1, 3–6] depend on a number of hand-labeled gold standard images. However, this type of approach presents two main problems. First, as noted by Hoover *et al.* [7] and cited by Fraz *et al.* [2] there is significant disagreement in the identification of vessels, even amongst expert observers. Secondly, not all problems are clearly labeled, for example there are uncertainties in the boundary of a vessel, especially in the small vessels. Also, vessels near pathology are difficult to label [7].

On the other hand, unsupervised vessel segmentation [7–16] is performed without training data or hand labeled ground truths. Such algorithms are able to reveal correlations, patterns, regularities or categories in the data samples. The ability to segment without hand labeled images allows to operate at a wide range of conditions and with retinal images obtained from different cameras.

Retinal vessel segmentation is a process that in most cases consists in enhancement of the vessels combined with a segmentation method. Many methods for unsupervised retinal vessel segmentation rely on information from vessel enhancement filters [7, 8, 10, 14, 17]. Popular filters are: matched filter [10], Frangi’s filter [18], and Gabor Wavelet filter [5]. Each filter responds differently to a vessel pixel. The combination of various filter responses in order to improve the segmentation results, is the main motivation of this work.

The idea is to weigh several individual filters and combine them in order to obtain an enhanced image that outperforms each one of them. A necessary condition for a combined filter to be more accurate than any of its individual members is that the result of each individual filter presents a high diversity [19]. In this work, an accurate vessel enhancement filter is one that enhances the pixels of the vessels and suppresses the intensity of the pixels belonging to the background.

Here, we propose an efficient unsupervised method for automatic retinal vessel segmentation that combines three vessel enhancement filters: matched filter [10], Frangi’s filter [18], and Gabor Wavelet filter [5]. These filters were chosen because of their individual performance and because they are based on different concepts, increasing the diversity of the filtering results. Two approaches are used to perform the filter combination: weighted mean and median ranking. The procedure is based on the combination of specific filters for the enhancement of vessels in the images, which serves as input to the segmentation methods. The main advantage of the proposed methods is that they are completely unsupervised. In addition, the approaches combine different filters that yield better results than using a single filter.

## Proposed Method

**Fig 1:**
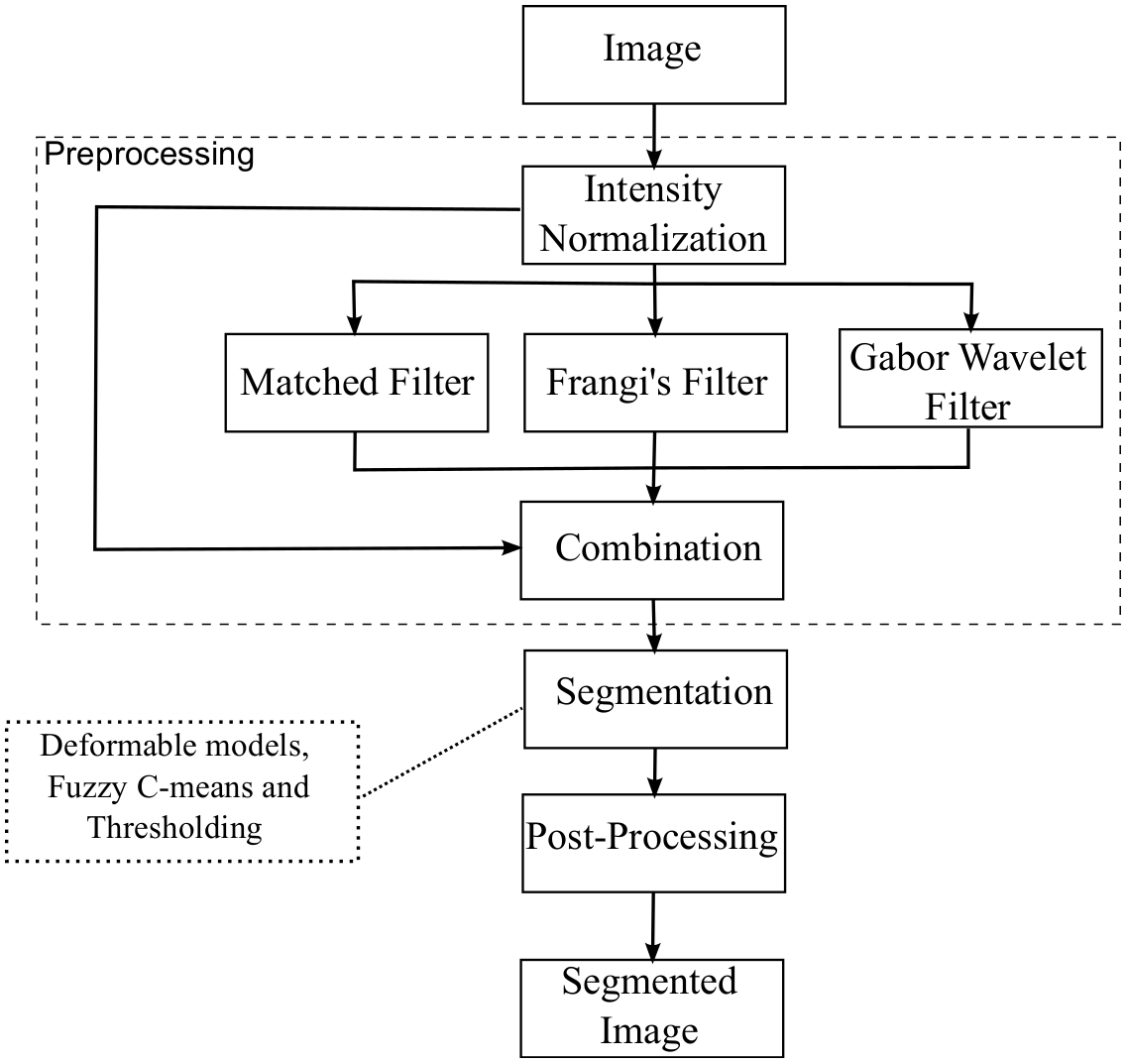
The proposed method. The method is composed of three different steps consisting of a combination of different filters and a segmentation method.

The proposed method for filter combination is divided into three steps, as shown in Fig 1. After intensity normalization of the vessel image, three specific filters for the enhancement of the blood vessels images are combined: matched filter [10], Frangi’s filter [18], and Gabor Wavelet filter [5]. These filters were chosen because they are the most used in the literature in retinal vessel segmentation [5, 7, 8, 10, 14, 17, 18]. The filters are based on Gaussian functions, which ensures good responses to the vessel enhancement filters [10]. Moreover, they are fast and easy to implement. The image is then segmented and noise is removed in a post-processing step. In the segmentation step we use the Fuzzy C-means, ORSF and optimize threshold approaches.

### Intensity Normalization

The first stage of the proposed method for the segmentation of the retinal vessels using filter combination is the pre-processing. In this step, we are concerned with the characteristic problem of retinal images, such as low contrast between the vessels, image background, and illumination. From the RGB model, only the green channel is selected because it exhibits the best contrast between vessels and background while the red and blue channels tend to be very noisy [20]. Contrast stretching improves the contrast and illumination of the image. The algorithm requires two limits, a lower value *α*_1_ and a higher *α*_2_ (these limits are set 0 and 1, respectively). Each pixel *p* is scaled according to

$$\begin{array}{c} p_{out}=\left(p-\beta_{1}\right)\frac{\left(\alpha_{2}-\alpha_{1}\right)}{\left(\beta_{2}-\beta_{1}\right)}+\alpha_{1}, \end{array}$$ (1)

where *β*_1_ and *β*_2_ are the lowest and highest pixel values present in the image, respectively.

**Fig 2:**
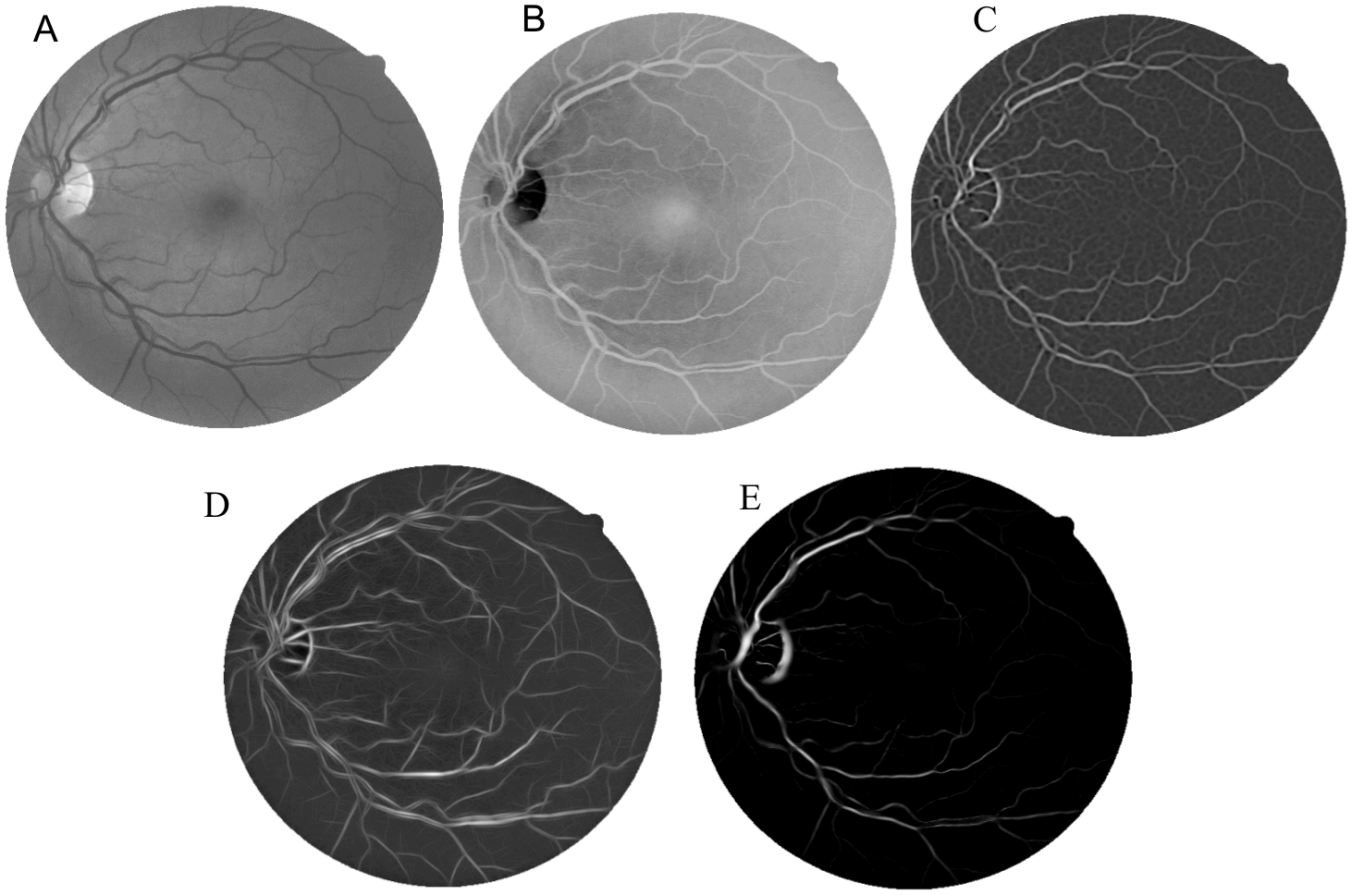
Results obtained from the filters. (A) Green channel, (B) Enhanced image using intensity normalization, (C) matched filter, (D) Frangi’s filter, and (E) Gabor Wavelet filter.

Matched filters extract the blood vessels by convolving the image with predefined filters that enhance the vascular structures features. The vessel intensity profile is symmetrical from a line passing through the center of the vessels. Gaussian models are the basis for the generation of filters for the vessels detection. Both matched [10] and Laplacian of Gaussian filters [8] are proper choices for the vessels detection. However, while the matched filter enhances the vessels using Gaussian filters with different orientations, the Laplacian of Gaussian is an isotropic filter. In this work, we used the matched filter as proposed by Chaudhuri *et al.* [10]. Fig 2C shows an enhanced image using this filter.

Frangi *et al.* [18] presented a technique that enhances the blood vessels in the images. A measure that describes the vascular structures is obtained from eigenvalues of the Hessian matrix. The enhancement process of this method considers the vessels as tubular structures. Since the vessels appear in different sizes, the process uses information obtained from a range of scales. Fig 2D shows an enhanced retinal image using Frangi’s filter.

Gabor Wavelet is the last filter used by the proposed method for the filter combination. Soares *et al.* [5] presented a method for the enhancement of the vessels using the Gabor Wavelet transform at various scales. The Gabor Wavelet is an elongated Gaussian function modulated by a complex exponential. Fig 2E illustrates the results from the Gabor Wavelet filter.

#### Filters Combinations

**Fig 3:**
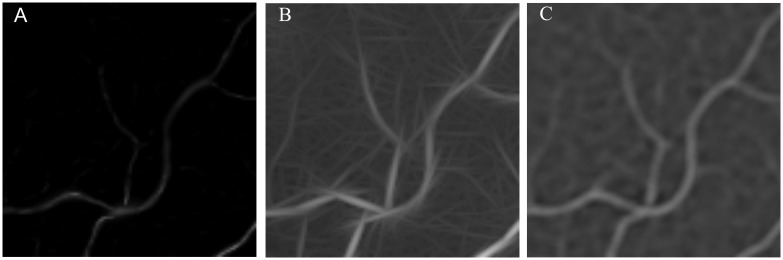
Regions of enhanced images. (A) Enhanced image using Frangi’s filter. (B) Enhanced image using Wavelet Gabor. (C) Enhanced image using matched filter.

The main motivation for filter combination is the complementarity that they provide. The Frangi’s filters, Gabor Wavelet and matched filter present different results and when these filters are combined, the overall result can be improved. Fig 3A–3C show a part of an enhanced image by the Frangi’s filter, Gabor Wavelet and Matched filter, respectively. The Wavelet Gabor and matched filters enhance the small vessels better, while Frangi’s filter is less sensitive to noise. In this subsection, the combination methods of weighted mean and median ranking are described.

#### Weighted Mean and Optimization

The combination approach using the weighted mean is similar to the method presented by Oliveira *et al.* [21]. It combines the filters through a weighted mean using Fuzzy C-means and deformable models. The weights were defined through many experiments. However, in this work we propose an approach for the automatic selection of weights, using Genetic Algorithms (GA) [22].

Let *G* and *W* be the enhanced images and non negative weights sets, respectively, where *g* ∈ *G* and *w* ∈ *W*. Enhanced images are normalized between 0 and 1. The weighted mean is then defined as follows:

$$\begin{array}{c} F=1-(\sum_{k=1}^{N}g_{k}w_{k}). \end{array}$$ (2)

where *N* is the number of enhanced images.

The set of enhanced images can include: Matched Filter (MF), Frangi’s filter (FR), Gabor Wavelet filter (GW), and Intensity Normalization (IN). The adjustment of the weights should enhance the small vessels while suppressing background structures. The weights are obtained using genetic algorithms constrained to:

$$\begin{array}{c} \sum_{k=1}^{N}w_{k}=1. \end{array}$$ (3)

**Fig 4:**
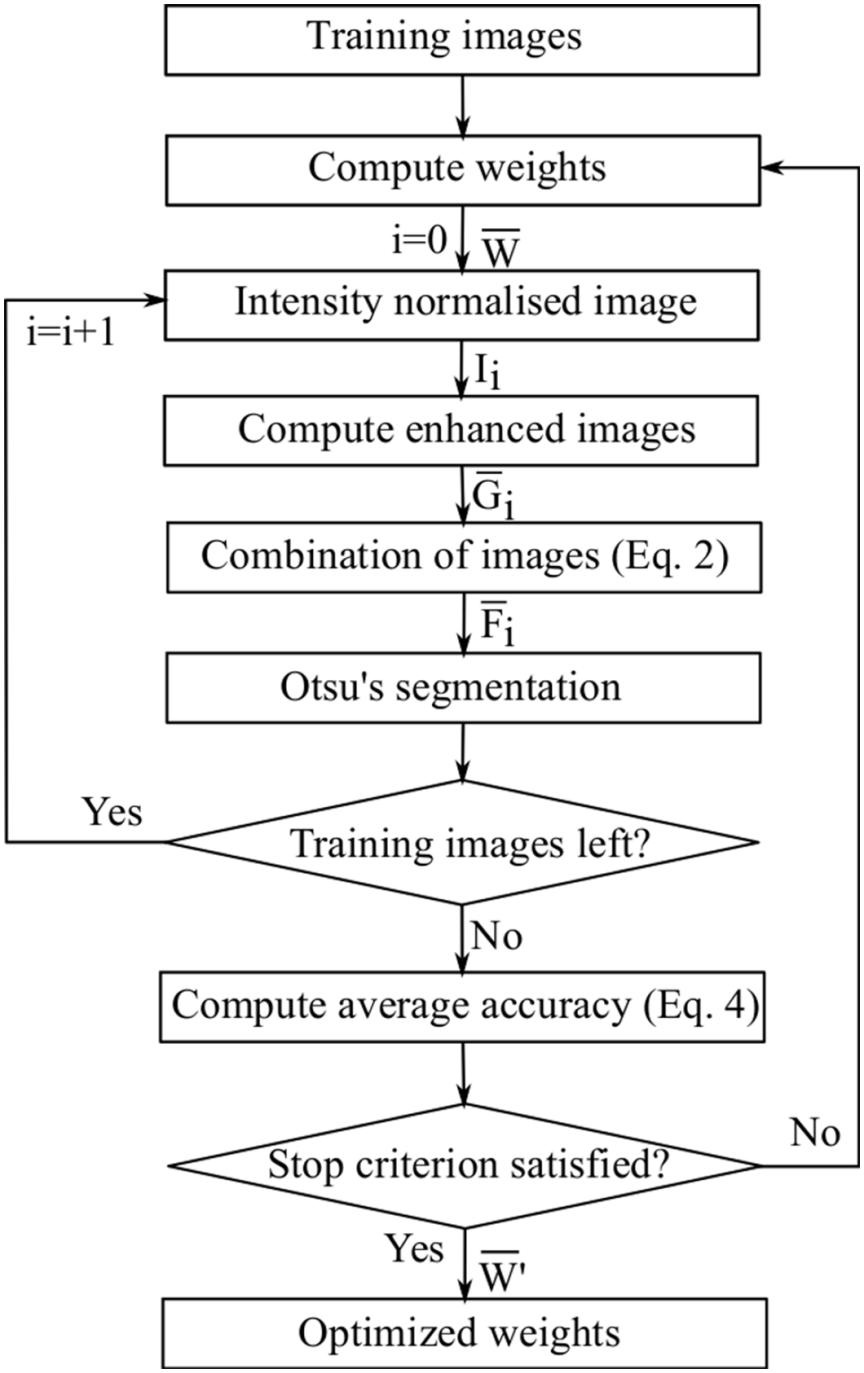
Steps to select the optimized weights using GA.

Genetic Algorithm is an optimization technique based on the principles of genetics and natural selection: from a population of individuals representing possible solutions, evolution is carried out by means of selection and reproduction. The desired solution is a set of parameters that minimizes the evaluation function (objective function). This function represents how much a given solution fits to the reference set delineated by a specialist. Fig 4 shows the steps to select the optimized weights using GA. These steps are executed for each possible combination of the filters set *τ*. Let *M* be the number of images in the training dataset. For each possible combination $\bar{\tau }\in \tau$ with $N_{\bar{\tau }}$ filters, the GA selects the weights $\bar{W}\in W$ at each iteration. Given a training image *I*_*i*_, *i* = 1, 2, …, *M*, the algorithm computes enhanced images subset ${\bar{G}}_{i}\in G_{i}$ and then obtains the combined image ${\bar{F}}_{i}$ according to Eq (2). Otsu’s algorithm segments the image ${\bar{F}}_{i}$. The algorithm evaluates the weights $\bar{W}$ according to the fitness function defined by Eq (4). This process is repeated until a stopping criterion has been reached. In the end, the algorithm selects the optimized weights ${\bar{W}}^{\prime }$ that minimizes the fitness function. The stopping criterion was defined as the maximum number of iterations without improvement of the current best solution, which was 50 iterations.

$$\begin{array}{c} f=1-\frac{1}{M}\sum_{i=1}^{M}ACC_{i}, \end{array}$$ (4)

where *ACC*_*i*_ is the accuracy of the image segmentation *i* defined by:

$$\begin{array}{c} ACC=\frac{\text{Number}\;\text{of}\;\text{correctly}\;\text{classified}\;\text{points}}{\text{Number}\;\text{of}\;\text{pixels}\;\text{inside}\;\text{FOV}\;}=\frac{TP+TN}{TN+TP+FN+FP}, \end{array}$$ (5)

in which *TP* stands for True Positive, *TN* (True Negative), *FP* (False Positive) and *FN* (False Negative). The ground truth databases used to calculate accuracy are described in [5] and [7]. For the segmentation procedure, we use the Otsu’s thresholding method [23]. In this work, genetic algorithm is used to find the best filter combination that enhances the pixels of the vessels and suppresses the intensity of the pixels belonging to the background, i.e facilitating the segmentation of the pixels into two classes: vessel or non-vessel. The main motivation to use Otsu as a segmentation algorithm in the process to select the optimized weights is because this algorithm shows an excellent trade-off between speed and effectiveness for the segmentation. It is a very fast method requiring no parameters to be set other than the number of classes, and yields optimal segmentation results if the classes are Gaussian distributed. However, as retinal images have many artifacts/noise, we use other, more sophisticated image segmentation methods to improve accuracy, as such fuzzy C-Means. For example, given a set of enhanced images obtained by the optimized weighted mean, the segmentation accuracy using the Otsu’s algorithm is 0.931, while the segmentation based on the fuzzy C-means algorithm presents accuracy equal to 0.94.

#### Median Ranking

Encouraged by the positive results involving system combination, Belkin *et al.* [24] performed two other experiments and reported the following observations: when multiple systems have incompatible scores, a combination method based on ranked outputs rather than the individual scores is the proper method for the combination. The enhanced images obtained by the filters presented in this paper have exactly this characteristic. Table 1 shows the Pearson’s correlation coefficients [25] of the retinal images enhanced by the filters. According to the table, Matched Filter (MF) and Frangi’s filter (FR) have higher linear correlation, since they produce more compatible scores. However, other filter pairs exhibit low or moderate correlation, meaning different incompatible scores.

**Table 1:** Pearson’s correlation coefficients for the enhancement filters.

**Filter**	**MF**	**FR**	**GW**	**IN**
**MF**	1	0.69	0.52	0.30
**FR**	0.69	1	0.58	0.18
**GW**	0.52	0.58	1	0.26
**IN**	0.30	0.18	0.26	1

In this work, we normalize the enhanced images obtained by the filters and then combine the normalized images using the median ranking. For each filtered image, the pixels are ordered from high to low according to the intensity of each pixel. High intensities usually indicate more influential features. Let *r*_*k*_*p*__ be the position in the rank of the pixel *p* in the image enhanced by filter *k*, *k* = 1, 2, …, *N*, where *N* is the number of filters. The pixel with higher intensity of image enhanced by filter *k* is assigned rank by *r*_*k*_*p*__ = *top*, the runner-up, *r*_*k*_*p*__ = *top* − 1, and so on, where *top* is the total number of pixels. We calculate the median ranking for each pixel *p* as:

$$\begin{array}{c} \bar{r_{p}}=med\left(r_{1_{p}},r_{2_{p}},\cdots ,r_{N_{p}}\right). \end{array}$$ (6)

**Fig 5:**
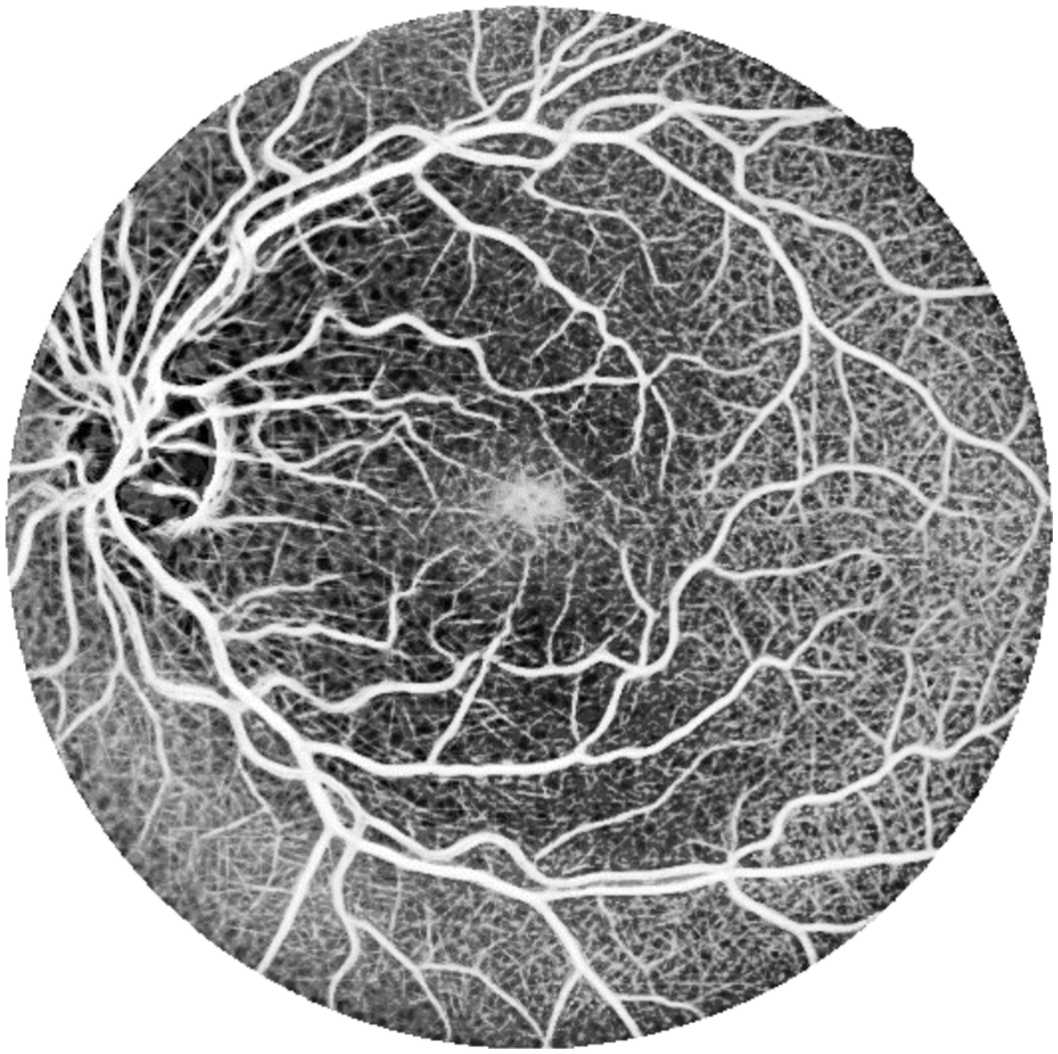
Enhanced image using median ranking.

Fig 5 shows an example of an enhanced image using median ranking.

### Segmentation Methods

In our filter combination system, after the enhanced images have been combined, we use different segmentation methods. The segmentation methods are related to the type of approach for filters combination. Enhanced images with median ranking are segmented with a simple threshold, which is obtained automatically using Genetic Algorithms (GA) on the training images of the Drive database and the six first images of the Stare database. In the case of the Drive database, the authors provided a training dataset with 20 images. For the Stare database, the total number of images was 20. We choose 6 training images that represent 30% of this dataset. This value was obtained as a compromise between a good representation in the training of the images and sufficient number of images for the testing. The fitness function is based on accuracy of the segmented images using the threshold obtained at each iteration of the GA, similarly as shown in Eq (3). Whereas, for the enhanced retinal images based on weighted mean, we used two approaches deformable models and fuzzy C-means algorithm, as described below. These segmentation methods were chosen based on their performance. In our deformable model based approach, we have extended the previous approach [26], by modifying the kernel function to account for the vessels orientation.

#### Deformable Models

Recently, image segmentation based on deformable models is considered one of the greatest successes in the area of image processing [26, 27]. The segmentation of medical images is one of the fields in which this application has proved to be very useful. Compared with other segmentation methods, an algorithm based on deformable models is more flexible and can be used for more complex operations.

In this paper, the deformable model is based on the Region-Scalable Fitting (RSF) Energy approach presented by [26]. This method is a variation of Chan and Vese model [27], described as an active contours approach to the Mumford-Shah problem [28]. The advantage of this approach is that it works well on images with non-homogeneous features, such as those presented in images of blood vessels. Furthermore, we propose a modification in order to make it more efficient in the segmentation of the retinal images.

The algorithm is based on local information intensities controllable in terms of scale. The fitting energy is defined by an active contour and two fitting functions that locally approximate the image intensities on both sides of the contour. The RSF computes local intensity fitting energy on the two sides of the active contour due to Gaussian kernel function with a scale parameter.

Let Ω ⊂ ℜ^2^ be the image domain, *I* : Ω → ℜ a given gray level image, *C* is defined as a closed contour in the image domain Ω and *x* a point in Ω. The energy functional proposed to minimize is [26]:

$$\begin{array}{c} \xi \left(C,f_{1}\left(x\right),f_{2}\left(x\right)\right)=\sum_{i=1}^{2}\lambda_{i}\int \left(\int_{\Omega_{i}}K_{\sigma }\left(x-y\right){|I\left(y\right)-f_{i}\left(x\right)|}^{2}dy\right)dx+\nu \left|C\right| \end{array}$$ (7)

where *y* are points in a neighborhood of *x*, Ω_1_ = outside(C) and Ω_2_ = inside(C), *ν*, *λ*_1_ and *λ*_2_ are positive constants, and *f*_1_(*x*) and *f*_2_(*x*) are functions that approximate image intensities in Ω_1_ and Ω_2_, respectively. The kernel function *K* is defined as:

$$\begin{array}{c} K_{\sigma }\left(u\right)=\frac{1}{2\pi \sigma^{2}}e^{-\frac{u^{2}}{2\sigma^{2}}} \end{array}$$ (8)

where *σ* is the parameter that controls the scale and *u* = *x* − *y*.

In this approach, we propose a change from the original method. Because the intensity profile of the vessel is symmetrical in relation to a line passing in the center of the vessels and the vessels are present in the image in several orientations [10], we propose a new kernel function to take into account the vessels orientation. Let $\bar{p}=\left[x,y\right]$ be a discrete point in the kernel and *θ*_*i*_ be an orientation. The rotation matrix is defined by:

$$\begin{array}{c} \bar{R_{i}}=\left[\begin{array}{cc} cos\;\theta_{i} & -sin\;\theta_{i}\\ sin\;\theta_{i} & cos\;\theta_{i} \end{array}\right] \end{array}$$ (9)

and the rotated point is given by $\bar{p_{i}}=\left[u,v\right]=\bar{p}{\bar{R}}_{i}^{T}$. The Gaussian filters are obtained using the following function:

$$\begin{array}{c} K_{i}\left(x,y\right)=e^{-\frac{u^{2}}{2\sigma^{2}}}\;\forall \;\bar{p}\in Z, \end{array}$$ (10)

where *Z* is a neighborhood such that *Z* = {(*u*, *v*), |*u*| ≤ 3*σ*, |*v*| ≤ *δ*/2} and *δ* is the length of the filter and the direction of the blood vessel is aligned along the y-axis. By varying the direction in 15° steps, 12 Gaussian filters are generated. This function is similar to that described by [10]. The maximum response for each point of the image is retained.

The initialization of the contour is performed by the method proposed by Sofka and Stewart [29], which detects the vessels centerlines. Once the initial contour is computed, the algorithm uses an image obtained according to Eq (2). The result of this operation is a binary image. This approach is referred to as the minimization of Oriented Region-Scalable Fitting energy (ORSF).

#### Segmentations using Fuzzy C-means

The fuzzy C-means clustering method is the second approach used to segment an enhanced image by the combination using weighted mean. Clustering is the task of assembling a set of objects into groups so that objects in the same group are more similar to each other than those in other groups. The fuzzy C-means algorithm (FCM), developed by Dunn [30] and improved by Bezdek [31], is one of the most common approaches to solve a clustering problem. The FCM is a clustering method that allows that one element can belong to one or more groups to a certain degree. The FCM is often used in pattern recognition and image segmentation.

In this paper, we use the FCM algorithm to segment the image *F* formed by the combination of filters. We observe that the non-vascular structures and the background have a lot of variation in gray level. Therefore, we choose *c* groups: *c* − 1 serve to group the pixels of non-vessels and only one group is used to group the vessels pixels.

### Post-processing

This step is necessary to remove noise and small regions that do not present elongated structures, since they are not part of the vessel. First, the blobs are identified in the binary segmented image. Then, for each blob, a value that indicates how the blob is elongated is calculated by the following equation:

$$\begin{array}{c} E=\frac{P^{2}}{4\pi a}, \end{array}$$ (11)

where *P* is the perimeter and *a* is the object area. In case *E* is less than a threshold *l*, which is obtained experimentally, in this case 2, the object is disposed.

## Experiments and Results

### Image Databases

**Fig 6:**
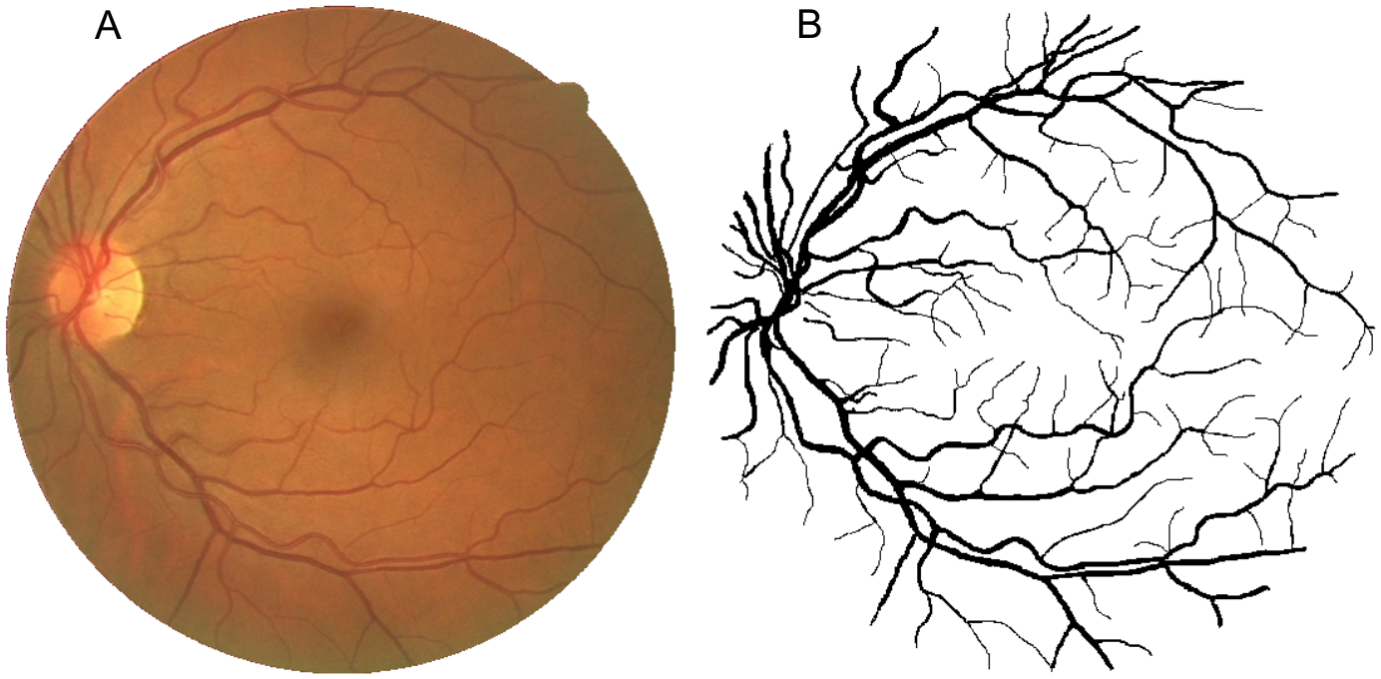
Example of an image of Drive database. (A) Image of Drive database and (B) its respective manual segmentation.

The proposed segmentation method using a combination of filters is tested with two public retinal image databases: Drive [3] and Stare [7]. The Drive database consists of 20 training images and 20 test images. The test set only contains four images with pathologies. The images were captured using a Canon CR5 non-mydriatic camera with a field of view having 45 degrees. The field of view (FOV) of each image is circular with a diameter of approximately 540 pixels. For each image, a mask image is provided that delineates the FOV and also two manual segmentations of the vessels. Each image was captured at 768 × 584 pixels and use 8 bits per color channel in the RGB model. Fig 6 illustrates an image of the test set with its respective manual segmentation.

**Fig 7:**
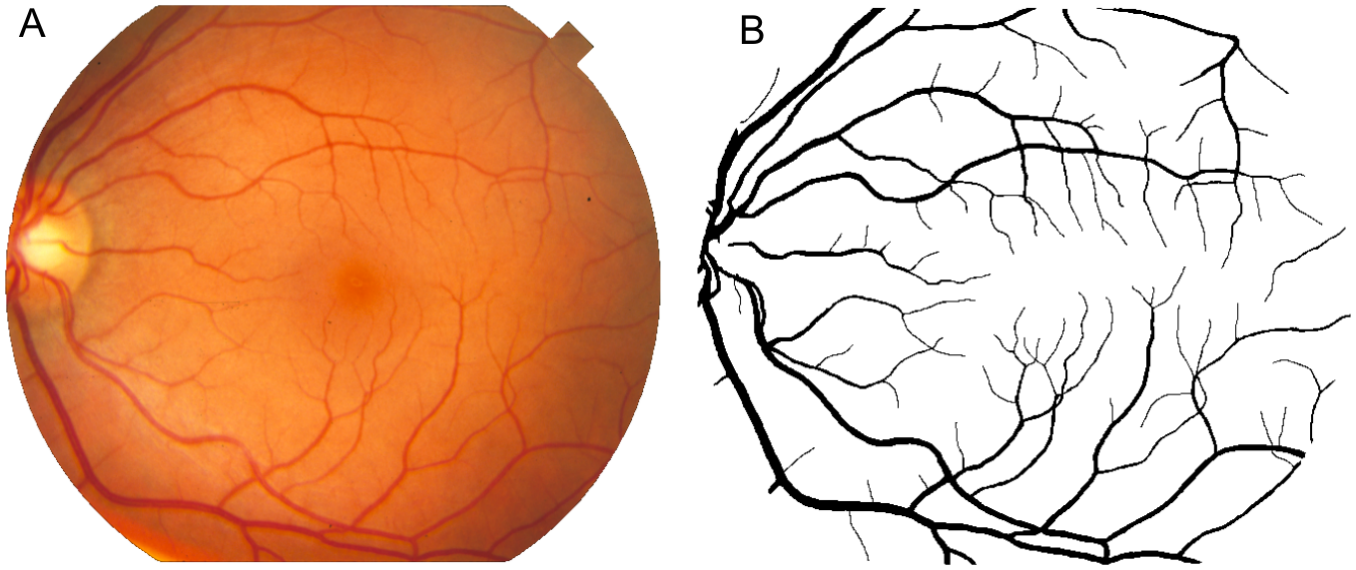
Example of an image of Stare database. (A) Image of Stare database and (B) its respective manual segmentation.

The Stare database contains 20 retinal images, including ten images with pathologies. The images were captured using a TopCon TRV-50 fundus camera at 35^°^ FOV. The images were digitalized to 700 × 605 pixels and 8 bits for each color channel. The database also provides hand-labeled images as the ground truth for vessel segmentation so that the algorithms can be evaluated. Fig 7 illustrates an image of this database and its corresponding manual segmentation.

### Evaluation Metrics

The proposed method is evaluated both qualitatively and quantitatively. The quality of image segmentation is based on visual observations. Quantitative evaluation is done by analyzing error images, which are obtained from the difference in the segmentation of one of the proposed methods in relation to the manual segmentation. The quantitative evaluation of the image segmentation is not a straightforward procedure. There are different quality criteria described in the literature. Accuracy rate (ACC), True Positive Rate (TPR), False Positive Rate (FPR), Area Under the ROC Curve (AUC), Connectivity-Area-Length (CAL) and Matthews Correlation Coefficient (MCC). We will now describe these measures in detail.

Accuracy is the degree of conformity of a measured quantity to its actual (true) value. It is the most widely metric used to quantify the performance of the segmentation methods from images of retinal vessels. The accuracy of the retinal vessel segmentation is defined in Eq (5). The maximum value for the accuracy is 1.0. The true positive rate (TPR) is the sensitivity, whereas the false positive rate (FPR) is associated with the specificity. A ROC curve is a plot of the true positive rate versus the false positive rate by varying the threshold on the probability map. The closer the curve approaches the top left corner, the better the performance of the system. The Area Under the Curve (AUC), which is 1 for a optimum system, is a single measure to quantify this behavior. The AUC is the unique measure calculated using the enhanced image (gray level). All other measures are based on the binary image, where the operating point is determined by the segmentation algorithm.

The Matthews Correlation Coefficient (MCC) is a measure of the quality of a binary classification [32]. It is a measure suitable even when the samples in the two classes are unbalanced, as is the case of the blood vessels in retinal images, where the number of non-vessel pixels is higher than the number of vessel pixels. The MCC values vary between -1 and +1. A coefficient of +1 represents a perfect prediction, 0 no better than random prediction and -1 indicates a total discordance between the obtained classification and the ground-truth. This measure was introduced in 1975 by Matthews [33] and the formula is defined as follows:

$$\begin{array}{c} MCC=\frac{TP\times TN-FP+FN}{\sqrt{\left(TP+FP)(TP+FN)(TN+FP)(TN+FN\right)}}. \end{array}$$ (12)

Gegúndez-Arias *et al.* [34] proposed a function, denoted by CAL, which evaluates the vessel connectivity, area and length in a segmented image in comparison with those in a reference-standard image. This measure analyzes the segmentation considering vascularity as a tree-like connected structure with specific anatomical features and not an individual pixel-to pixel comparison. The function is given by:

$$\begin{array}{c} f(C,A,L)=C\times A\times L\equiv CAL. \end{array}$$ (13)

Connectivity *C* is defined by Eq (14). *S* is defined as the segmentation to be evaluated and *S*_*G*_ as the reference image:

$$\begin{array}{c} C\left(S,S_{G}\right)=1-min\left(1,\frac{|\#c\left(S_{G}\right)-\#c\left(S\right)|}{\#(S_{G})}\right), \end{array}$$ (14)

where #*c*(*S*) and #*c*(*S*_*G*_) are the number of connected components in *S* and *S*_*G*_, respectively, and #(*S*_*G*_) denotes the cardinality of *S*_*G*_.

The degree of overlapping areas between the *S*_*G*_ and *S* is calculated by:

$$\begin{array}{c} A\left(S,S_{G}\right)=\frac{\#(\left(\delta_{\alpha }\left(S\right)\cap S_{G}\right)\cup \left(S\cap \delta_{\alpha }\left(S_{G}\right)\right))}{\#(S\cup S_{G})}, \end{array}$$ (15)

where *δ*_*α*_ is a morphological operator using a disk with radius equal to *α*.

The factor measuring the degree of coincidence in terms of length is given by:

$$\begin{array}{c} L\left(S,S_{G}\right)=\frac{\#(\left(\varphi \left(S\right)\cap \delta_{\beta }\left(S_{G}\right)\right)\cup \left(\delta_{\beta }\left(S\right)\cap \varphi \left(S_{G}\right)\right))}{\#(\varphi \left(S\right)\cup \varphi \left(S_{G}\right))}, \end{array}$$ (16)

where *φ* is the result of a skeletonization algorithm and *δ*_*β*_ is a morphological dilation using a disk of radius *β*. As suggested by Gegúndez-Arias *et al.* [34], *α* and *β* values were set to 2.

### Choice of the Parameters

The weighted mean combination filters use Genetic Algorithm to select the best weights for the Matched Filter (MF), Frangi (FR), Gabor Wavelet (GW), and Intensity Normalization (IN), as explained in the Section Weighted Mean and Optimization. Table 2 shows the accuracy obtained by varying all filters and contrast-enhanced image in combination with the weighted mean. According to the table, for the Drive database we obtain accuracy greater than 89% for any combination of filters and segmentation method. The combination using all filters and image contrast shows the best result for this database. For the individual filters, the matched filter presented the best performance. The combination using the three filters showed a lower accuracy rate than the combination of matched filters, Gabor Wavelet filter and intensity normalization. However, for the Stare database, the combinations using MF & GW and MF & FR & GW produced better results using FCM and ORSF methods, respectively.

**Table 2:** Accuracy obtained by varying all filters and contrast-enhanced image in the combination using weighted mean.

Combinations	Drive		Stare	
	FCM	OSRF	FCM	OSFR
MF & FR & GW	0.9379	0.9320	0.9392	**0.9429**
MF & FR & IN	0.9340	0.9317	0.9115	0.9402
MF & GW & IN	0.9383	0.9329	0.9442	0.9378
FR & GW & IN	0.9345	0.9185	0.9121	0.9355
MF & FR	0.9297	0.9304	0.9262	0.9344
MF & GW	0.9340	0.9329	**0.9446**	0.9391
MF & IN	0.9346	0.9317	0.9442	0.9371
FR & GW	0.9313	0.9061	0.9264	0.9347
FR & IN	0.8993	0.9186	0.9262	0.9344
All	**0.9402**	**0.9356**	0.8736	0.9407
MF	0.9309	0.9302	0.9422	0.9370
FR	0.8961	0.9158	0.9262	0.9343
GW	0.9306	0.9007	0.8117	0.9077
IN	0.5287	0.9207	0.6271	0.9329

The algorithm is configured having the following parameters obtained experimentally for the Drive database: *λ*_1_ = 3, *λ*_2_ = 3, *ν* = 130. According to the results of Table 2, the combination chosen is ALL filters, which has weights equal to 0.6187, 0.0105, 0.2580, and 0.1128 for filters MF, FR, GW, and IN, respectively. The ORSF for Stare database has the following configuration, obtained experimentally *λ*_1_ = 2, *λ*_2_ = 2, *ν* = 130. The parameter *l* of the post processing step in both database is equal to 3. The best weights selected by the genetic algorithm are 0.5117, 0.3834, 0.1045 for filters MF, FR and GW.

For the fuzzy C-means algorithm, it is necessary to define the number of classes *c*. For the Drive and Stare database two classes are used. For both databases, value of *l* of the post processing step is equal to 2, determined experimentally. In the Drive database, the optimized weights 0.6187, 0.0105, 0.2580, and 0.1128 are defined for the filters MF, FR, GW, and IN, respectively. Whereas for the Stare database, the algorithm uses the optimized weights 0.9058 and 0.0941 for MF and GW, respectively.

For the proposed approach using median ranking, the retinal images from the Drive database are segmented using a threshold equal to 0.8602. Whereas for the Stare database, we use the threshold equal to 0.8808. These thresholds were optimized using Genetic Algorithms (GA) on the training images of the Drive database and the six first images of the Stare database. The fitness function is based on the accuracy of the segmented images using the threshold obtained at each iteration of the GA, similarly as shown in Eq (3).

### Analysis of the Experiments

**Fig 8:**
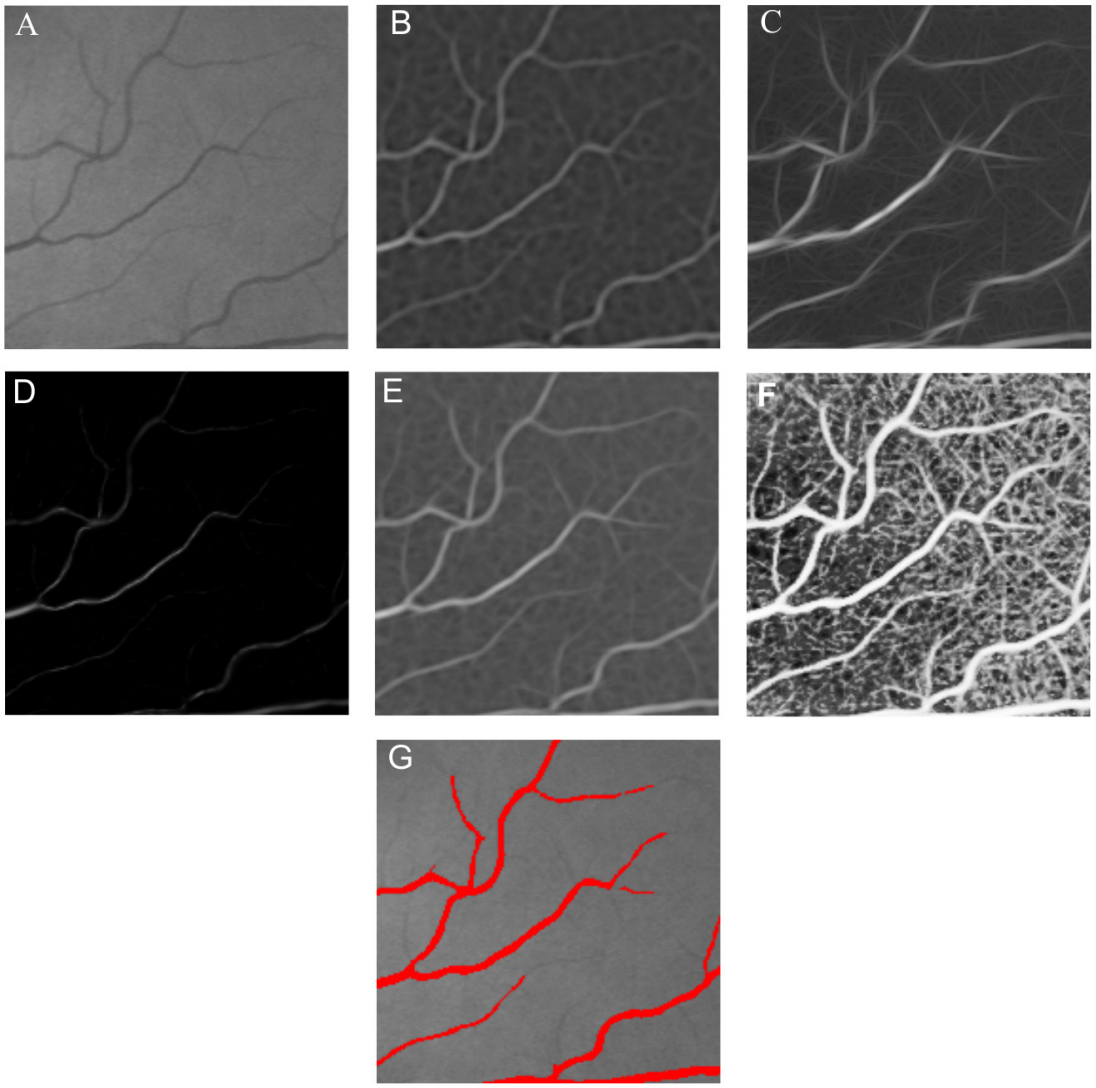
Response obtained from the filters and our combination methods. (A) Green channel, (B) matched filter, (C) Gabor Wavelet filter, (D) Frangi’s filter, (E) Weigthed mean, (F) Median ranking and (G) Segmented image overlaid on the original image.

This paper proposes two approaches to combine vessel enhancing filters, named weighted mean and median ranking. Fig 8 shows part of a retinal image, green channel (A), and the enhanced version using matched filter (B), Gabor Wavelet filters (C), Frangi’s filters (D), combination using weighted mean (E) and median ranking (F). Notice that the combination of weighted mean and median ranking give good results for thin vessels, whereas the combination using weighted mean is more robust to the background of the image that are not vessels than the combination using median ranking. Although the approach based on median ranking enhances both noises and thin vessels, the final result is a good segmentation, as can be appreciated from Fig 8G, because the pixel values in the blood vessels are significantly larger than the noise level, requiring only a simple threshold to segment an retinal image.

**Fig 9:**
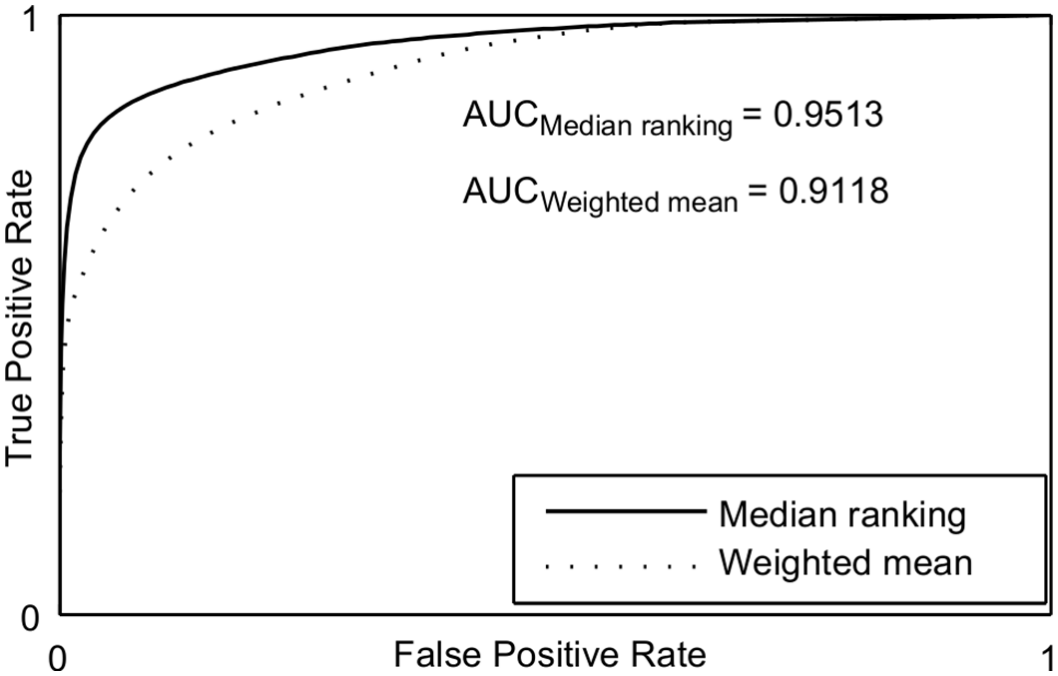
ROC curves of the proposed approaches for the DRIVE database.

**Fig 10:**
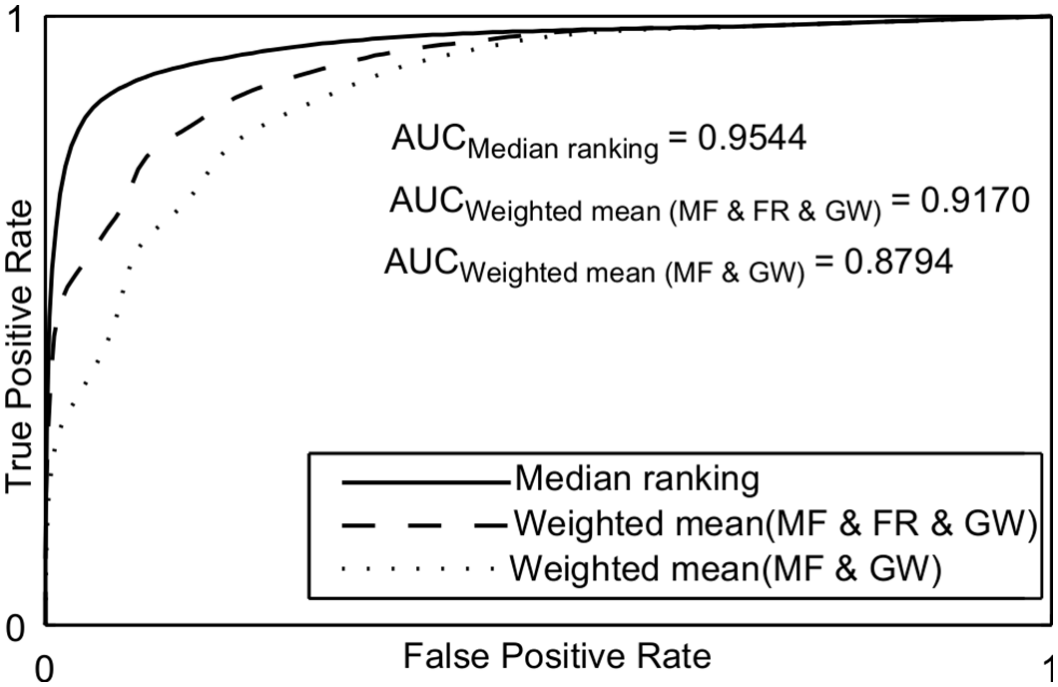
ROC curves of the proposed approaches for the Stare database.

**Fig 11:**
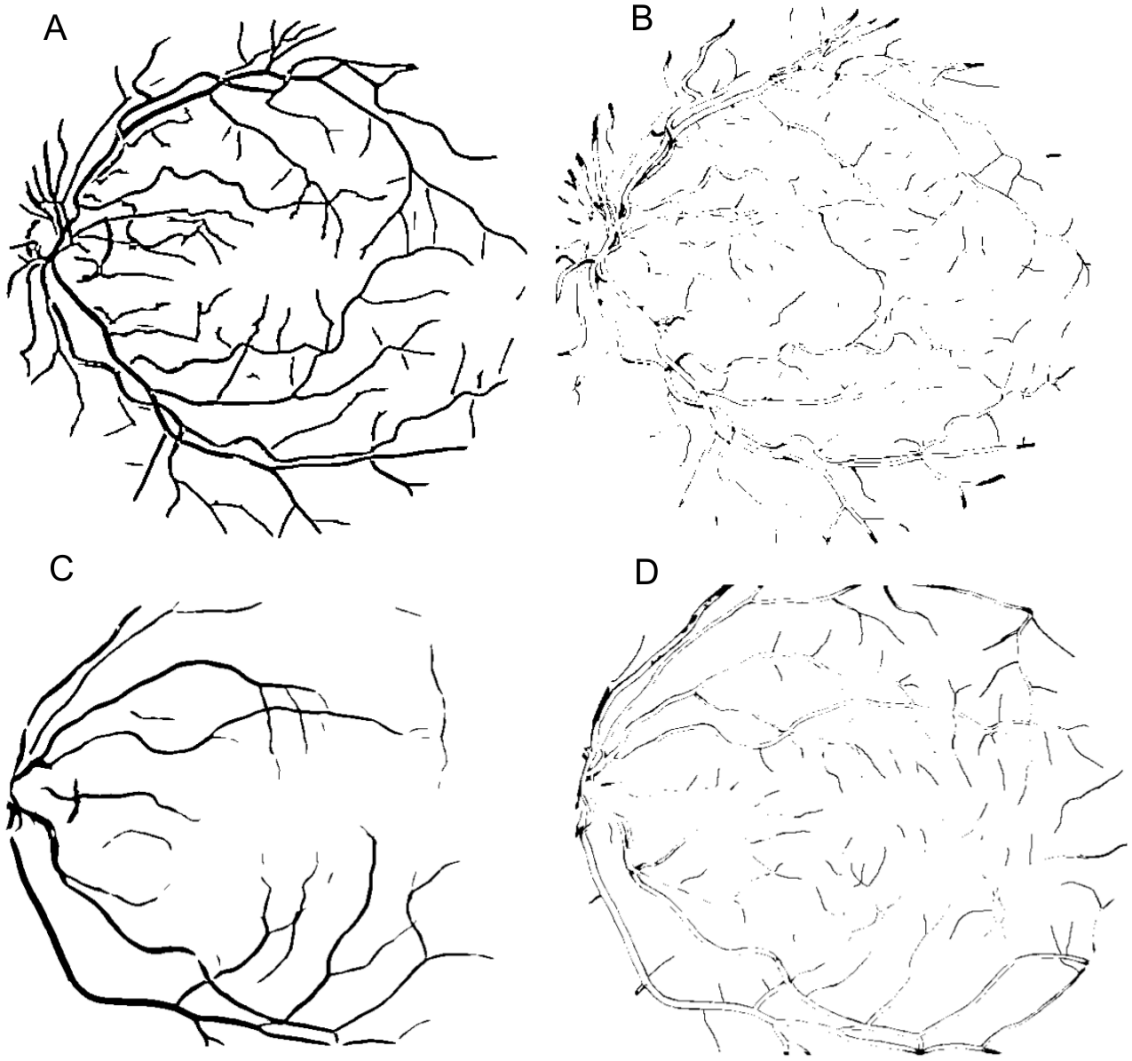
Result using ORSF. (A) Segmented image of the Drive database (Original image shown in Fig 6). (B) Error image represented in black color referring to the image (A). (C) Segmented image of the Stare database (Original image shown in Fig 7). (D) Error image represented in black color referring to the image (C).

**Fig 12:**
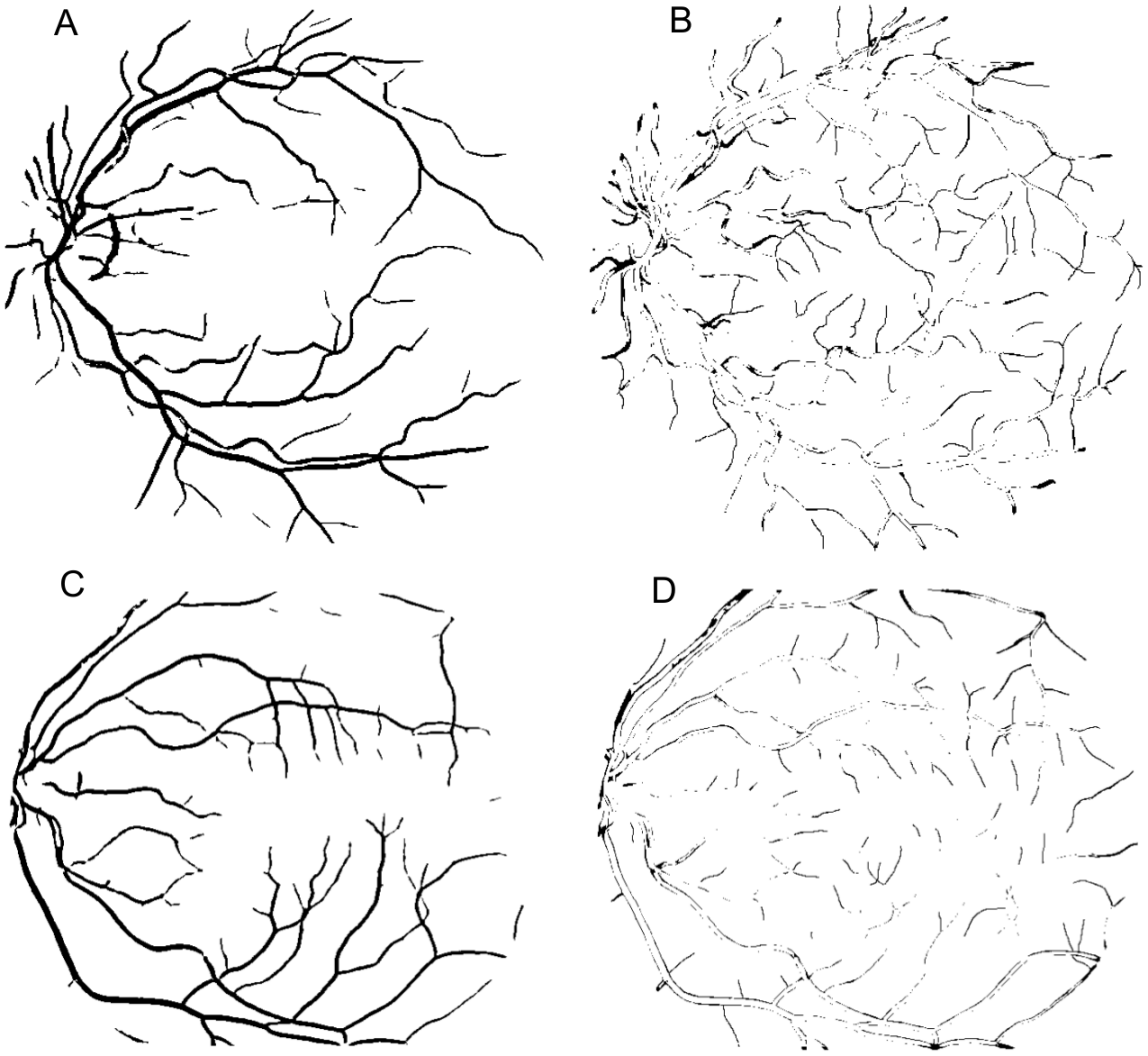
Result using FCM. (A) Segmented image of the Drive database (Original image shown in Fig 6). (B) Error image represented in black color referring to the image (A). (C) Segmented image of the Stare database (Original image shown in Fig 7). (D) Error image represented in black color referring to the image (C).

**Fig 13:**
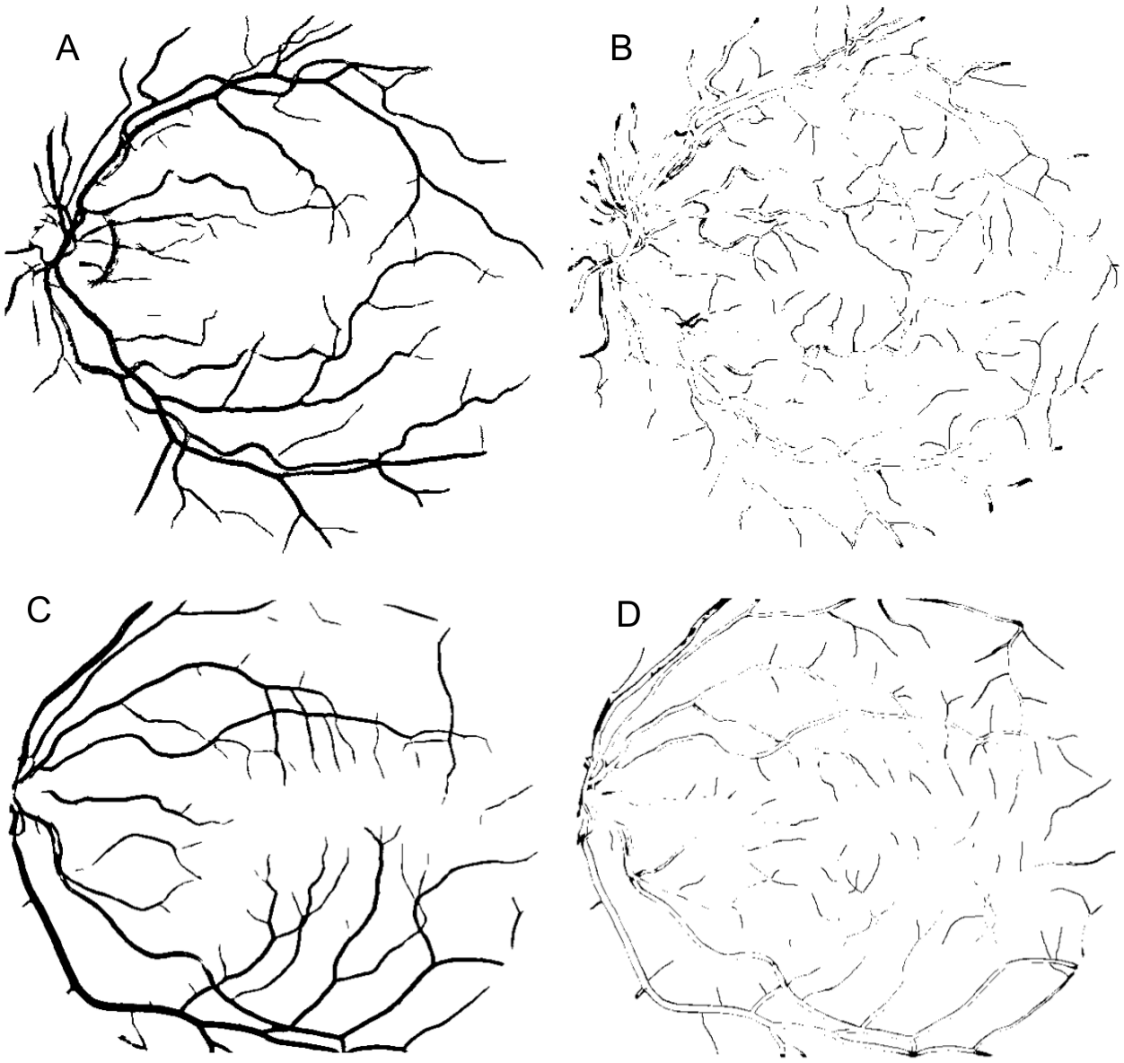
Result using median ranking. (A) Segmented image of the Drive database (Original image shown in Fig 6). (B) Error image represented in black color referring to the image (A). (C) Segmented image of the Stare database (Original image shown in Fig 7). (D) Error image represented in black color referring to the image (C).

Figs 9 and 10 show the Receiver Operating Characteristic (ROC) curves [35] of the proposed approaches for both retinal image databases. The proposed approach using median ranking has significantly better area under the ROC curve. Table 3 shows the performance of the proposed approaches in relation to supervised and unsupervised methods for the Drive database test set. The bold values represent the results of the proposed methods and the italic values represent the best rates. Comparing all methods, the proposed approach presents values very close to the best supervised segmentation methods. The accuracy rate of Fraz’s supervised [2] and Zhao [36] unsupervised approach are the two highest values in the table. The proposed approach using median ranking has the fifth best accuracy and a value very close to both other ones. For the unsupervised methods, the median ranking has the third highest performance measures compared to other methods. The weighted mean approach using FCM and ORSF have performance measures inferior to supervised methods. However, they are superior to most unsupervised methods, where the use of FCM provides better results than using ORSF. Similarly, the median ranking has the second highest MCC value among the unsupervised methods. Comparing the three unsupervised methods presented in this paper, they all present compatible result for each metric. This can be seen in Figs 11(A), 12(A) and 13(A), where the result of segmentation using weighted mean with ORSF can be considered more tree-like connected structure than the others. The character “-” means that the method was not implemented and the value metric was not described by the authors in their paper. We do not present all the values in Tables 3 and 4 because for some methods the authors did not present the values for the AUC, TPR, FPR or CAL metrics and the codes are not available.

**Table 3:** Performance results compared with others methods for the Drive database.

Methods	ACC	TPR	FPR	AUC	CAL	MCC
***Supervised***						
2nd Observer	0.9473	0.7761	0.0275	-	0.8485	0.7601
Marin *et al.* [4]	0.9452	0.7067	0.0275	0.9588	-	-
Soares *et al.* [5]	0.9466	0.7283	0.0212	0.9614	*0.75068*	*0.74885*
Niemeijer *et al.* [1]	0.9416	0.6898	0.0304	0.9294	0.68567	0.71779
Staal *et al.* [3]	0.9442	0.6780	*0.0170*	0.9520	0.71563	0.73228
Fraz *et al.* [37]	*0.9480*	*0.7406*	0.0193	*0.9747*	-	-
***Unsupervised***						
**Weighted mean using ORSF**	**0.9356**	**0.7988**	**0.0475**	**0.9118**	***0.70209***	**0.69402**
**Weighted mean using FCM**	**0.9402**	***0.9106***	**0.0569**	**0.9118**	**0.55289**	**0.70417**
**Median ranking**	**0.9464**	**0.8644**	**0.0444**	**0.9513**	**0.68343**	**0.74253**
Jiang *et al.* [13]	0.9212	-	-	0.9327	-	-
Mendonça *et al.* [15]	0.9463	0.7315	*0.0219*	-	-	-
Zana *et al.* [8]	0.9377	-	-	-	0.63949	0.72355
Martinez-Perez *et al.* [14]	0.9344	0.7246	0.0345	-	0.57846	0.66151
Al-diri *et al.* [9]	0.9258	-	-	-	-
Chaudhuri *et al.* [10]	0.8773	-	-	0.7878	0.24814	0.42088
Zhang *et al.* [17]	0.9382	0.712	0.0276	0.7878	-	-
Li *et al.* [12]	0.9310	0.6455	0.0337	-	-	-
Bankhead *et al.* [11]	0.9371	0.7027	0.0283	0.7816	0.62534	0.7056
Yin *et al.* [16]	0.9475	0.7556	0.0344	-	-	-
George *et al.* [38]	0.9442	0.7655	0.0296	*0.9614*	-	*0.7475*
Zhao *et al.* [36]	*0.953*	0.744	0.0220	0.861	-	-

**Table 4:** Performance results compared with others methods for the Stare database.

Methods	ACC	TPR	FPR	AUC	CAL	MCC
**Supervised**						
2*nd* Observer	0.9354	0.8949	0.0610	-	0.6504	0.7224
Marin *et al.* [4]	0.9526	0.6944	*0.0181*	*0.9769*	-	-
Soares *et al.* [5]	0.9480	0.7165	0.0252	0.9671	0.58928	*0.58305*
Staal *et al.* [3]	0.9516	0.6970	0.0190	0.9614	-	-
Fraz *et al.* [37]	*0.9534*	*0.7548*	0.0237	0.9768	-	-
**Unsupervised**						
**Weighted mean using ORSF**	**0.9429**	**0.8377**	**0.0491**	**0.9170**	**0.53154**	**0.64838**
**Weighted mean using FCM**	**0.9446**	**0.8049**	**0.0408**	**0.8794**	**0.58461**	**0.68583**
**Median ranking**	***0.9532***	**0.8254**	**0.0353**	**0.9544**	***0.64687***	**0.72458**
Jiang *et al.* [13]	0.9009	-	-	0.9298	-	-
Mendonça *et al.* [15]	0.9440	0.6996	0.0270	-	-	-
Zhang *et al.* [17]	0.9484	0.7177	*0.0247*	-	-	-
Martinez-Perez *et al.* [14]	0.9410	0.7506	0.0431	-	-	-
Hoover *et al.* [7]	0.9267	0.6751	0.0433	0.7590	0.54037	0.61474
Li *et al.* [12]	0.9407	0.7191	0.0313	-	-	-
George *et al.* [38]	0.9497	0.7716	0.0299	*0.9563*	-	*0.7335*
Zhao *et al.* [36]	0.951	*0.786*	0.0250	0.881	-	-

Table 4 presents the vessel segmentation accuracy rate (ACC), true positive rate (TPR), false positive rate (FPR) and the three other measurements AUC, CAL and MCC for the Stare database. The proposed unsupervised median ranking method, comparing ACC values, shows the second best performance among all the other methods including three supervised learning methods and it even outperforms the human observer. Among the unsupervised methods, the approach of [36][38] and [17] have greater accuracy than the proposed approaches with weighted mean using FCM and ORSF. However, the proposed approaches with weighted mean exhibit better performance than the methods [14] and [13], and the use of FCM has higher accuracy than using ORSF. Also the median ranking method shows high values for the AUC, CAL and MCC when compared to Soares et al. [5] and Hoover et al. [7].

In both tables, the proposed approaches show high values of TPR, which means a high degree of accuracy in the vessels, especially the median ranking approach. However, the high value of FPR means that this accuracy in the vessels is accompanied by an error above the average of non-vessels pixels classified as vessels. For this reason metrics such as AUC, CAL and MCC are important to be analyzed.

Analyzing the capacity of generalization of the approaches for combination, the weighted mean is more sensitive to characteristics of the image database than median ranking because the weights were optimized according to the training images. However, considering approach weighted mean, the generalization of the optimized combination for the Drive database is higher than the optimal combination for Stare database. The accuracy result for the Stare database using the best combination for the Drive database with FCM and ORSF algorithms, given by the combination *All*, are 0.8600 and 0.9245 for FCM and ORSF, respectively. Similarly, the FCM and OSFR algorithms using the best combination for the Stare database in Drive database images, given by MF & GW and MF & FR & GW, show accuracy values of 0.9332 for FCM and 0.9232 for ORSF. Although the accuracies are below of the best values for each database, it is noticed that when we apply the best combination to database Stare in the Drive database, the result is closer to the highest accuracy values in Table 2. The fact that the Stare database present various pathological images, makes it difficult for any segmentation method that uses an optimal combination of the Drive database to obtain a good result in the Stare database. This becomes clear when we tested only the images without pathologies of the Stare database with the best combination obtained in the Drive database. In this case, the FCM and ORSF algorithms obtained accuracies equal to 0.9491 and 0.9362, respectively.

In terms of qualitative results, the Figs 11 and 12 show a segmented image of each database for approach using weighted mean with FCM and ORSF, respectively. Fig 13 shows the qualitative result for the median ranking approach.

## Discussion and Conclusions

This work presents a framework to segment blood vessels in retinal images using unsupervised approaches. The combination of filters have relatively better responses compared to a single filter. We use two approaches for the combination of the filters: median ranking and weighted mean. The proposed methods benefit from the different responses to the same pixel.

The approaches were evaluated and compared with some of the best performing methods in the area. According to [5], one of the difficulties found in the evaluation of the segmentation methods of retinal vessel is the establishment of a reliable metric from manually labeled images. Both Drive and Stare databases have 20 labeled images for testing, which were manually obtained by an expert. Therefore, the ground truth is also subjective to this expert. Ideally, each image should have several manual segmentations performed by different experts. Moreover, a precision measure for pixel-by-pixel classification, such as the sensitivity and the specificity, raises some questions. A pixel that is correctly classified does not necessarily correspond to a good vessels segmentation when considering the context. Since most of the image is defined as background, if the whole image is set as background, we would obtain a good value for the accuracy. However, this would not provide any image segmentation. Metrics such as AUC, CAL and MCC are of interest because they try to overcome this difficulty. They are presently not widely used, specially CAL and MCC. However we think they are important, because they consider the quality of the segmentation process in a more global way.

However, only 20 images or 40, if we take both databases in consideration, may not be enough to perform a statistical test reliably. Soares *et al.* [5] suggested the use of experts for the tests realization.

The proposed approaches are very effective for the retinal vessels segmentation. In quantitative terms, the approach using the median ranking has superior performance than using the weighted mean approach. Moreover, it presents rates higher than the unsupervised methods, described in this work. This approach has better accuracy rate than the supervised methods for the Stare database and performance close to the best method for the Drive database. The weighted mean approach using FCM has better results in terms of accuracy than the ORSF method.

A qualitative visual evaluation is still important, allowing to define advantages and disadvantages of the method, despite being subjective [5]. Even though quantitative results were good, especially in terms of accuracy, in the qualitative evaluation the approaches presented some problems in the segmentation, such as in the thin vessels and in areas close to the borders. These are still some of the challenges in vessel segmentation. In recent years, several articles have been published proposing different methods to segment retinal vessel images. However, an important question that still needs a better clarification is how to best compare the different methods, since ACC by itself is a limited measurement because most of the image can be considered as background. This is an issue not only limited to retinal vessel segmentation but also to any image segmentation problem that have subjective ground truth. For the retinal vessel segmentation problem, we made use of the measurements AUC, CAL and MCC to improve the comparison.

### Algorithm availability

The MATLAB implementation of our algorithm is included as supporting information (S1 File). For sample data, the Drive and Stare databases are available at http://www.isi.uu.nl/Research/Databases/DRIVE/ and http://www.ces.clemson.edu/~ahoover/stare/ respectively.

## Supporting Information

S1 File MATLAB implementation of our algorithm. (ZIP)
